# The London Panel Committee: Tuesday's Decisions

**Published:** 1914-10-17

**Authors:** 


### The London Panel Committee: Tuesday's Decisions.

A special meeting of the London Panel Committee was held on Tuesday last, when the Chairman, Dr. H. J. Cardale, announced that the subscriptions received towards the fund opened after the last meeting had reached the sum of £530, and a motor ambulance costing £550 had been purchased for presentation to the British Red Cross Society. The Panel Committee recently accepted the principle that the local Medical Committee for London should be represented upon the Committee. It was stated, however, that difficulty had been experienced in finding members who were able to serve, and in order to complete the membership of the Committee the following practitioners on the panel were co-opted:—Drs. A. E. Dodson, P. D. Pywell, and C. K. Toland.

In regard to the sixty-five practitioners of whose prescriptions the Pharmaceutical Committee has demanded an investigation under Article 40 of the Medical Benefit Regulations, 1913, on the ground that the supply of drugs ordered is in excess of what is reasonably necessary, it was proposed that, as a matter of convenience, the Panel Committee should delegate its duties in this respect to the Pharmacy Sub-Committee. The question was raised whether such power could legally be delegated, but it was stated that the Insurance Commissioners had ruled that it could.

Dr. R. J. Farman urged that the question was one of urgency, because another batch of sixty-five complaints was ready, and 120 more would follow. Whichever Committee undertook the work, it would probably have to sit until Christmas. Dr. Hogarth said that many of the prescriptions which would come under examination carried their own condemnation without doubt or qualification. The Chairman explained that the procedure would be that the Committee would finally approve or disapprove the Sub-Committee's findings, and each practitioner would have the right, if he desired, to appeal to the Commissioners.

Members asked for some examples of the prescriptions that had been given, and the Chairman mentioned the following:—The ordering of urotropine under its own name; the ordering of cough mixtures, quinine and iron mixtures, and bismuth mixtures in 16 oz. quantities; 4 oz. of liquid extract or ergot in one prescription; 8 lb. of cod-liver oil in one week; prescriptions which should last five days being repeated day after day; a prescription consisting only of spirits of peppermint to the value of 3s. 7d. with the direction, "add 6 oz."; ordering a 16 oz. mixture, and in two days ordering a different or the same mixture.

### Breaking Doctors' Windows.

Questions were asked as to what practitioners should do in cases where patients alleged that they had dropped the bottles of medicine, or stated that the medicine was too sour or to sweet, and asked for another mixture. The Chairman thought practitioners should have sufficient force of character to refuse such applicants, whereupon it was rejoined that in some districts doctors' windows would be broken.

The Committee agreed that the Sub-Committee should hear the evidence for and against each practitioner, and report to the Panel Committee as proposed.

It was reported that the Insurance Committee had sent a letter to each practitioner on the panel asking that the use of the formula "Rep. mist." might be discontinued. A letter of protest was ordered to be sent to the Insurance Committee on the ground that the issue of the circular ignored the fact that at a conference between representatives of the Panel and Pharmaceutical Committees a compromise was agreed upon whereby the formula "Rep. mist." might be used, provided the repeat prescriptions were given in the same calendar month as the original one, and the name and address of the patient were given in each case.

